# Detecting fixation on a target using time-frequency distributions of a retinal birefringence scanning signal

**DOI:** 10.1186/1475-925X-12-41

**Published:** 2013-05-13

**Authors:** Boris Gramatikov

**Affiliations:** 1 Laboratory of Ophthalmic Optics, Krieger Children’s Eye Center, Pediatric Ophthalmology and Adult Strabismus, Wilmer Ophthalmological Institute, Johns Hopkins University School of Medicine, Rm. 233, 600 N. Wolfe St, Baltimore, Maryland, USA

**Keywords:** Retinal birefringence scanning, Time-frequency distribution, Continuous wavelet transform, Amblyopia, Strabismus, Eye alignment

## Abstract

**Background:**

The fovea, which is the most sensitive part of the retina, is known to have birefringent properties, i.e. it changes the polarization state of light upon reflection. Existing devices use this property to obtain information on the orientation of the fovea and the direction of gaze. Such devices employ specific frequency components that appear during moments of fixation on a target. To detect them, previous methods have used solely the power spectrum of the Fast Fourier Transform (FFT), which, unfortunately, is an integral method, and does not give information as to where exactly the events of interest occur. With very young patients who are not cooperative enough, this presents a problem, because central fixation may be present only during very short-lasting episodes, and can easily be missed by the FFT.

**Method:**

This paper presents a method for detecting short-lasting moments of central fixation in existing devices for retinal birefringence scanning, with the goal of a reliable detection of eye alignment. Signal analysis is based on the Continuous Wavelet Transform (CWT), which reliably localizes such events in the time-frequency plane. Even though the characteristic frequencies are not always strongly expressed due to possible artifacts, simple topological analysis of the time-frequency distribution can detect fixation reliably.

**Results:**

In all six subjects tested, the CWT allowed precise identification of both frequency components. Moreover, in four of these subjects, episodes of intermittent but definitely present central fixation were detectable, similar to those in Figure 4. A simple FFT is likely to treat them as borderline cases, or entirely miss them, depending on the thresholds used.

**Conclusion:**

Joint time-frequency analysis is a powerful tool in the detection of eye alignment, even in a noisy environment. The method is applicable to similar situations, where short-lasting diagnostic events need to be detected in time series acquired by means of scanning some substrate along a specific path.

## Introduction

**Figure 1:**
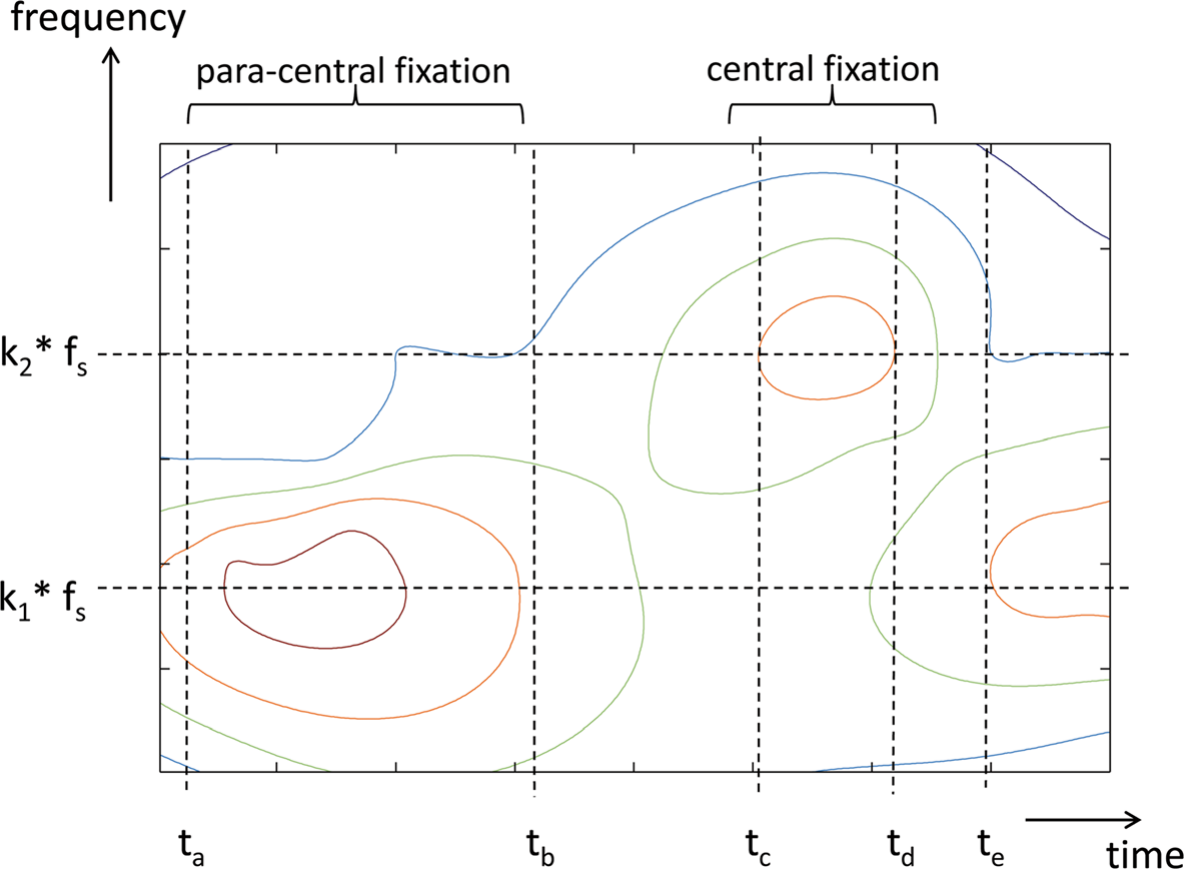
**Time-frequency distribution of signals, produced by a retinal birefringence scanning system.** Central fixation is characterized by the appearance of frequency component *k*_2_*f_*s*_.

The fovea, which is the most sensitive part of the retina, is known to have birefringent properties, i.e. it changes the polarization state of light upon reflection. The main cause for this birefringence are the Henle fibers surrounding the fovea, arranged in a radially symmetric pattern. When illuminated or scanned with polarized light, the Henle fiber layer produces the macular polarization cross or “bow tie”, which is a windmill shaped pattern centered about the fovea [1-3]. The strength and contrast of the polarization cross and the orientation of its bright parts depend on the orientation and degree of polarization of the light striking the retina, which is a function of both the instrumentation and the individual corneal properties [4,5]. In recent years, the birefringent properties of the human eye have been used to identify the position of the fovea and the direction of gaze. This allows for one to check for eye alignment and strabismus (cross-sightedness), a risk factor for amblyopia (called also “lazy eye”), which can potentially lead to a loss of sight in the affected eye. Screening techniques for amblyopia have been reported that are based on the birefringence signal derived from foveal circular scanning [6-8]. In this approach, a signal *s(t)* consisting of several frequency components (*f*_*1*_*= k*_1_*f_*s*_*, f*_*2*_*= k*_2_*f_*s*_*, f*_*3*_*= k*_3_*f_*s*_, etc.) is produced, where each frequency is a multiple of the scanning frequency *f*_*s*_. Some frequencies prevail during central fixation, while others appear at para-central fixation. The constants *k*_*1*_ , *k*_*2*_, *k*_*3,*_ etc. depend on the opto-mechanical design. Figure 1 shows the time-frequency distribution of such signals, produced by a system which generates two signals. Let us assume that the eye does not fixate on the target in the time interval [*t*_*a*_ … *t*_*b*_], and fixates during [*t*_*c*_ … *t*_*d*_]. Consequently, in the time-frequency plane, we observe concentration of power at frequency *k*_1_*f_*s*_ in the timeframe [*t*_*a*_ … *t*_*b*_], and then shift of the dominating frequency to *k*_2_*f_*s*_ during [*t*_*c*_ … *t*_*d*_]. At time *t*_*e*_ the eye loses fixation again, and consequently, the emphasis is shifted back to *f*_*1*_*= k*_1_*f_*s*_. In the simplest case, *f*_*2*_*= 2f*_*s*_ is produced during central fixation, while *f*_*1*_*= f*_*s*_ prevails during off-central fixation, i.e. *k*_*1*_*=* 1 and *k*_*2*_*=* 2 [6]. Existing instruments (i.e. [7]), acquire consecutive epochs of *s(t),* with gaps between them, during which an FFT is performed. A problem with this approach is that the FFT power spectrum is a global measure, i.e., it provides information on how much of *f*_*1*_ and *f*_*2*_ are represented in the whole epoch analyzed, but it does not provide information on exactly where these frequencies appear and for how long. With less-cooperative pediatric patients, important short-lasting moments of central fixation *(f*_*2*_*)* may easily be hidden behind large low-frequency *(f*_*1*_*)* components, and missed. Analyzing short time intervals is desirable, in order to increase temporal resolution, but this is where the FFT becomes prone to noise and loses spectral resolution. There thus remains a need for improved methods for detecting central fixation.

## Method

**Figure 2:**
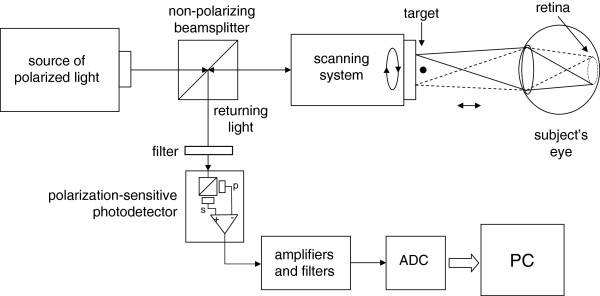
**A simplified block diagram of a device for retinal birefringence scanning.** The system measures changes in the polarization state of light caused by the retina.

The method proposed here assumes that an opto-mechanical apparatus already exists, which enables the acquisition of a birefringence signal from a circular retinal scan, where the scanning circle is positioned around the expected position of the fovea during fixation on a stationary target, appearing optically in the center of the scanning circle. Such systems have been developed and described elsewhere [6-10]. The general design of such a device is shown on Figure 2. In short, the system uses a low-power laser beam of vertically polarized near-infrared light, to scan the retina around the presumed center of the fovea and the polarization cross. The returning light is diverted to a polarization sensor, consisting of a polarizing beamsplitter and two photodetectors – one for the vertically polarized component *s* , and one for the horizontally polarized component *p*. The difference of the two gives a measure of the polarization state of light, called also *S*_*1*_ and being the second component of the full four-component Stokes vector [11]. The signal is amplified and filtered, to remove frequency components outside the region of interest, and finally digitized. The background, comprising the optical noise, is removed prior to any signal processing. This paper focuses on a design producing *f*_*2*_*= 2f*_*s*_ during central fixation and *f*_*1*_*= f*_*s*_ during off-central fixation (*k*_*1*_*=* 1, *k*_*2*_*=* 2) as reported in [7]. Scanning signals were recorded from six subjects after obtaining written informed consent, following a protocol approved by the Institutional Review Board (IRB) and in compliance with the Helsinki Declaration. Personal identification data was not collected, and the signals collected were not used for identification purposes. From each subject, 12 records were acquired, each 400 ms long, for both the right eye (RE) and the left eye (LE). The sampling frequency was 5 kHz, while the scanning frequency was 96 rounds per second.

The problem of detecting short-lasting episodes of central fixation, characterized by the appearance of *f*_*2*_*= 2f*_*s*_, can be successfully solved by using time-frequency distributions (TFD) obtained by means of the Continuous Wavelet Transform (CWT). It allows excellent localization in both time- and frequency domains and is computationally efficient. If the signal to be analyzed is *s(t)* and *g(t)* is the analyzing wavelet, the CWT is defined as a convolution of the type:

(1) $$W\left(\tau ,a\right)=\frac{1}{\sqrt{a}}\int s\left(t\right)g*\left(\frac{t-\tau }{a}\right)\mathrm{dt}$$

where * denotes complex conjugate , *a* - the scale (dilation), and *τ* - a time shift. The wavelet *g(t)* and the distribution *W*(*τ*,*a*) are complex-valued [12-17]. The constant 1/ is used for energy normalization. The analyzing wavelet satisfies the following conditions:

(2) $$s\left(t\right)=\frac{1}{c}\int_{g-\infty }^{\infty }\int_{a>0}^{\infty }W\left(\tau ,a\right)\frac{1}{\sqrt{a}}g\left(\frac{t-\tau }{a}\right)\frac{1}{a^{2}}\mathrm{dad\tau}$$

where *c*_*g*_ is a constant that depends only on *g(t)* and *a* is positive. For an analytic wavelet this constant should be positive and convergent

(3) $$c_{g}=\int_{0}^{\infty }\frac{{\left|G\left(\omega \right)\right|}^{2}}{\omega }\mathrm{d\omega}<\infty$$

which in turn imposes an admissibility condition on *g(t)*. For a real-valued wavelet, the integrals from both -∞ to 0 and 0 to +∞ should exist and be greater than zero. Admissible wavelets have no zero frequency contribution, or, what amounts to the same, they are of zero mean, or equivalently *G(ω) =* 0 for *ω*=0:

(4) $$\int_{-\infty }^{+\infty }g\left(t\right)\mathrm{dt}=0$$

(a) Belong to **L**^2^ (**R**), i.e. be *square integrable* (be of finite energy),

(b) Be *analytic* (*G(ω) =* 0 for *ω* < 0 ) and thus be complex-valued,

(c) Be *admissible*. This condition was shown to enable invertibility of the transform:

An appropriate choice for an analyzing wavelet is the *admissible* complex-valued wavelet of Morlet [12,18] comprising a modulated Gaussian function of optimal time-frequency concentration:

(5) $$g_{m}\left(t\right)=e^{j\omega_{0}t}e^{-\frac{t^{2}}{2}}$$

**Figure 3:**
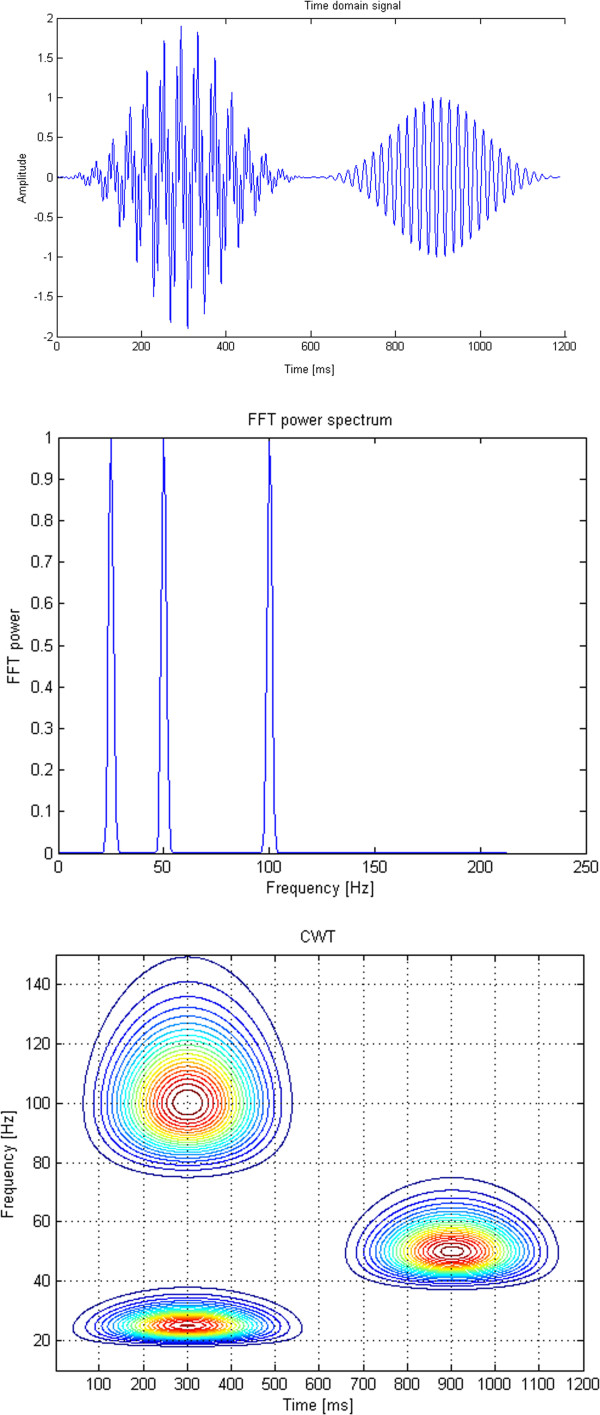
**Analysis of a surrogate signal *s(t) *containing two bursts of sinosoidal signals.** The first burst is a mixture of two sine waves (25 Hz and 100 Hz) occurring simultaneously, whereas the second burst, shifted in time, contains a sine wave of 50 Hz. FFT and CWT are performed on the same time signal.

As an example, Figure 3 shows a surrogate signal *s(t)* containing two bursts of sinosoidal signals. The first burst is a mixture of two sine waves (25 Hz and 100 Hz) occurring simultaneously, whereas the second burst, shifted in time, contains a sine wave of 50 Hz. Each of the two bursts was modulated by a three-term Blackman-Harris window:

(6) $$s\left(t\right)=W_{\mathrm{BH}}\left(t-t_{1}\right)\sum_{i=1}^{2}\sin\left(2\pi f_{i}t\right)+W_{\mathrm{BH}}\left(t-t_{2}\right)\sin\left(2\pi f_{3}t\right)$$

where *f*_*1*_*=* 25 Hz, *f*_*2*_ =100 Hz, *f*_*3*_*=* 50 Hz and each modulation window of duration T and starting times respectively t_1_ =0 ms and t_2_*=* 600 ms, was calculated according to:

(7) $$\begin{array}{ccc} W_{\mathrm{BH}}\left(t\right)= & 0.42-0.50\cos\left(2\mathrm{\pi t}\right)+0.08\cos\left(4\mathrm{\pi t}\right) & \mathrm{for}\;0<t<T\\ W_{\mathrm{BH}}\left(t\right)= & 0 & \mathrm{for}\;t<0\;\mathrm{or}\;t>T \end{array}$$

The time domain signal is shown in the upper panel of Figure 3, whereas the FFT of the same signal is shown on the middle panel, and the time-frequency distribution calculated with the CWT (equations 1 and 5) is shown on the bottom panel as a contour plot. Notably, while the FFT does detect all three frequencies correctly in the frequency domain, it does not localize the time moments of the three bursts. The CWT, conversely, does localize all three events in both time and frequency.

## Results

**Figure 4:**
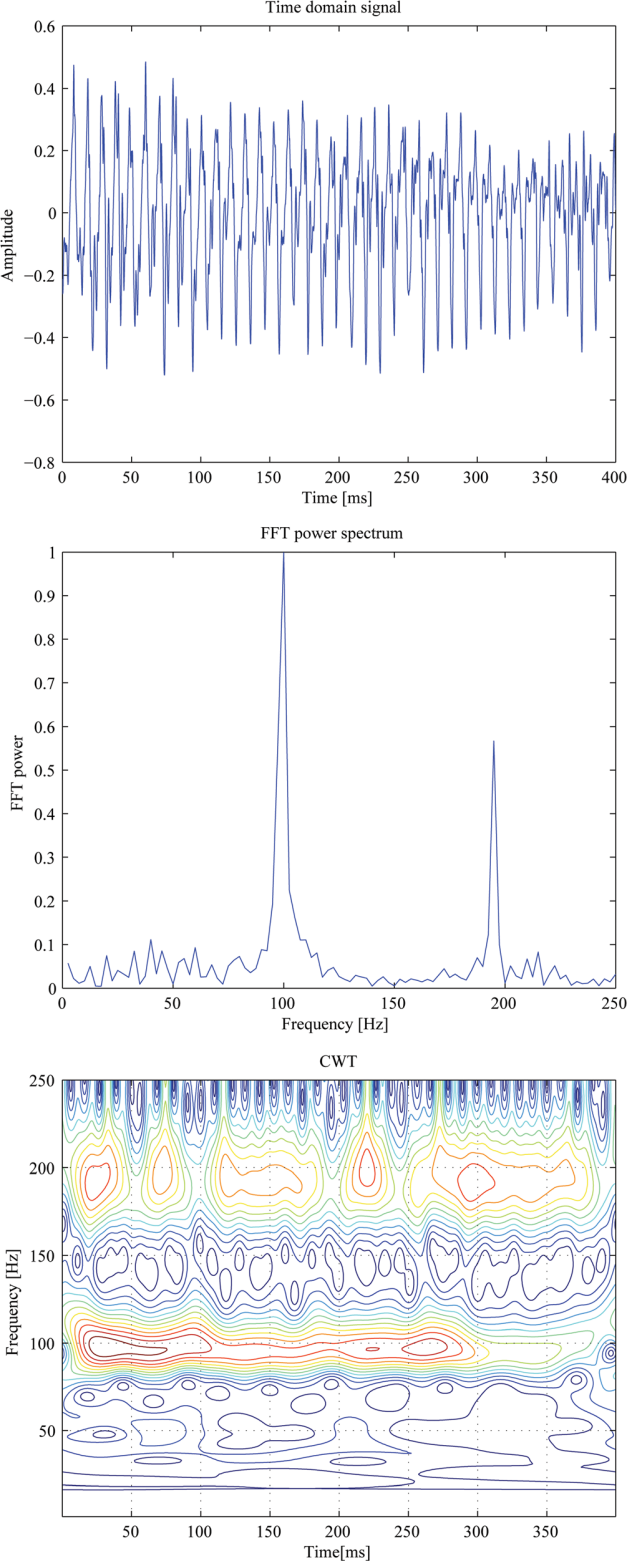
**Time-frequency distribution of a real patient data (right eye of a 7 year old boy).** The top panel shows the time domain scan signal *s(t).* The middle panel shows the FFT power of the same signal. The CWT (bottom panel) reveals short-lasting episodes of central fixation, with *f*_*2*_*=* 192 Hz.

For every epoch (T *=* 0.4 s) of the incoming signal, a time-frequency distribution was computed by means of the CWT (equations 1 and 5). Figure 4, top panel, shows the time domain scan signal *s(t)* (right eye of a 7 year old boy, *f*_*s*_*=* 96 Hz; *k*_*1*_*=* 1, *k*_*2*_*=* 2). The subject was trying to fixate on a target. The middle panel shows the FFT power of the same signal. Two main frequency components are present – one at 96 Hz (*f*_*1*_*= k*_1*_f_*s*_*=* 96 Hz), characteristic of para-central fixation, and one at 192 Hz (*f*_*2*_*= k*_2_*f_*s*_*=* 192 Hz), due to central fixation. Based on the FFT plot, one can easily decide that the prevailing frequency is *f*_*1*_ and therefore para-central fixation was prevailing during this record. With the CWT (bottom panel), episodes of *f*_*1*_ and *f*_*2*_ can easily be localized in time and in frequency dimensions simultaneously. A close look at the appearances of these components gives enough evidence to conclude that there were substantial short-lasting episodes of central fixation, manifested by intermittent appearance of components at *f*_*2*_*=* 192 Hz. They last between 30 ms and 100 ms each, and show up throughout the epoch. In the first 270 ms they exist side-by side with the para-central fixation components (*f*_*1*_*=* 96 Hz), which at times are of higher amplitude. One can easily see why the para-central components mask the central fixation components in the FFT. It should be mentioned here that sometimes remnants of the optical noise, not entirely eliminated by the background subtraction, appear at the scanning frequency *f*_*s*_*= f*_*1*_*=* 96 Hz, and can further mask the central fixation components in the FFT. Likewise, despite the polarization-killing properties of the human skin in general, some polarized portion of the light gets reflected by the face and returns into the optical system, reaching the sensors and giving rise to additional interference at *f*_*s*_*= f*_*1*_*=* 96 Hz. This emphasizes the importance of analyzing the signals in the time-frequency plane, which gives a better chance of separating signal from noise.

In all six test subjects, the CWT allowed precise identification of both frequency components. Moreover, in four of these subjects, episodes of intermittent but definitely present central fixation were detectable, similar to those in Figure 4. A simple FFT is likely to treat them as borderline cases, or entirely miss them, depending on the discrimination thresholds used. In one subject with stable central fixation, the frequency component *f*_*2*_*= k*_2_*f_*s*_*=* 192 Hz was strong and persistent when the subject was fixating on the target, and therefore both the FFT and CWT revealed frequency doubling. But the vast majority of recordings were a constantly changing mixture of the two spectral components. Depending on the time interval analyzed and the duration of central fixation episodes, the FFT was able to detect many episodes, usually the longer lasting ones (longer than 150 ms) and in the absence of noise, whereas the CWT *always* detected central fixation, even short instants lasting less than 60 ms. Uncompensated portions of the background noise did sometimes affect the *f*_*s*_*= f*_*1*_*=* 96 Hz signal, but never the central fixation frequency *f*_*2*_*= k*_2_*f_*s*_*=* 192 Hz.

## Discussion

This work is related mainly to methods aiming at detection of strabismus (cross-sightedness), eye alignment and amblyopia (“lazy eye”) in young test subjects, where patient cooperation is problematic and the detection of short-lasting episodes is important and even crucial in some cases. The robust application of the CWT makes it applicable to any type of optical systems acquiring and analyzing signals distinguishable in the joint time-frequency domain, especially in systems with abundant optical noise of origin different from that of the signals measured. A condition would be that the noise does not overlap in time and frequency simultaneously with the frequency and temporal location of the event. In addition to amblyopia, the technique described can be used for the detection of frequent and short-lasting losses of fixation, possibly indicative of nystagmus, attention-deficit-hyperactivity disorder (ADHD), autism, or other neuropsychologic disorders.

The CWT is not the only technique available for time-frequency expansion. The short-time Fourier transform, STFT, (also known as the *windowed Fourier transform*) localizes the signal in time- and frequency domain by modulating the time signal with a window function before performing the Fourier transform, to obtain the frequency content of the signal in the region of the window. As a rule, it is a compromise between time and frequency resolution; the wider the window, the higher the frequency resolution, at the cost of poorer time resolution, and vice versa [16]. Any attempt to increase the frequency resolution causes a larger window size and therefore a reduction in time resolution, and vice-versa. Also, in order to be able to analyze transients, overlapping windows need to be used, which can slow down analysis considerably, and acts like a low-pass filter in the time domain.

Another alternative, most widely used two or three decades ago, is the Wigner-Ville distribution (WVD). Its definition for time-frequency analysis is:

(8) $$W_{s}\left(t,f\right)=\int_{-\infty }^{\infty }s\left(t+\frac{\tau }{2}\right)s*\left(t-\tau /2\right)e^{-i2\pi \tau }\mathrm{d\tau}$$

where $i=\sqrt{-1}$ is the imaginary unit, and * denotes complex conjugation [19-22]. In essence, the WVD is the Fourier transform of the input signal’s autocorrelation function, i.e. the Fourier spectrum of the product between the signal and its delayed, time reversed copy, as a function of the delay. Unlike the short-time Fourier transform, the Wigner distribution function is not a linear transform. A cross term (“time beats”) occurs when there is more than one component in the input signal, analogous in time to frequency beats.

In order to reduce the cross term problem, many other transforms have been proposed, the most prominent one perhaps being the Cohen’s class distribution. The best known member of Cohen’s class distribution function is the Choi–Williams distribution function [22]. This distribution function adopts an exponential kernel to suppress the cross-term:

(9) $$C_{s}\left(t,f\right)=\int_{-\infty }^{\infty }\int_{-\infty }^{\infty }A_{s}\left(\eta ,\tau \right)\Phi \left(\eta ,\tau \right)e^{-i2\pi \left(\mathrm{\eta t}-\mathrm{\tau f}\right)}\mathrm{d\eta d\tau}$$

where

(10) $$A_{s}\left(\eta ,t\right)=\int_{-\infty }^{\infty }s\left(t+\frac{\tau }{2}\right)s*\left(t-\tau /2\right)e^{-i2\mathrm{\pi \eta t}}\mathrm{dt}$$

and the kernel function is

(11) $$\Phi \left(\eta ,\tau \right)=e^{-\alpha {\left(\eta \tau \right)}^{2}}$$

However, the kernel gain does not decrease along the η and τ axes in the ambiguity domain, and, consequently, the kernel function of the Choi–Williams distribution function can only filter out the cross-terms resulting from components away from the η and τ axes and away from the origin.

In summary, the CWT appears to be the tool of choice when time-frequency distributions are needed in order to detect different frequency components appearing simultaneously, or in different moments in time. This is particularly true of high frequency events of short duration and low amplitude (small scales), or longer-lasting low-frequency oscillations of higher amplitude (large scales), as is the case here.

There are some limitations of the optical and opto-mechanical hardware involved in this technology. Mechanical vibrations, optical back-reflections (reflections from reflective surfaces back to the sensors before actually the light reaches the eyes), multiple internal reflections not captured by the light traps, some birefringence caused by the beam splitters, and others, can all cause instrumental noise which translates into parasitic frequency components. Some of them may overlap with the central fixation frequency of interest and thus mask it, making a differentiation in the joint time-frequency domain difficult. Such artifacts can be avoided or moved away from the region of interest, by careful choice of the mechanical parameters (like scanning speed), optical design (i.e. spinning wave plates), or proper design of the analog electronics (i.e. filters).

Other limitations arise from the measurement principle. Because only about 1/1000 of the polarized light used for measuring is returned by the fovea, the distance between the measurement system and the eye cannot be increased much beyond 40 cm. Further, the room illumination should be dimmed, to prevent pupil constriction and reduction of the near-infrared light going through it. This not only decreases the signsl-to-noise ratio, but can cause patient discomfort, and is one more reason to aim for fast and reliable tests.

## Conclusion

The Continuous Wavelet Transform is superior to the FFT and most other TFD techniques in localizing fixation frequencies in both the time- and frequency domains. It is an excellent tool for precisely identifying central fixation in an uninterrupted manner, thus improving device reliability and shortening test time. When done in a binocular manner, i.e. for both eyes simultaneously, it allows the detection of short-lasting moments of eye alignment or misalignment, or even lack of attention to the target. Using modern digital signal processing hardware like digital signal processors, field programmable gate array (FPGA) logic, for example, the CWT can be performed in real time, and is expected to improve significantly detection sensitivity when testing uncooperative young subjects.

## Abbreviations

FFT: Fast fourier transform; CWT: Continuous wavelet transform; s(t): A signal consisting of several frequency components, produced by retinal scanning; fs: Scanning frequency; f1, ,f2, f3: Frequency components, produced by retinal scanning; k1, k2, k3: Constants, dependingf on the opto-mechanical design; [ta … tb], [tc … td]: Time intervals; RE: Right eye; LE: Left eye; TFD: Time-frequency distribution(s); g(t): An analyzing wavelet; *: Denotes complex conjugate; a: The scale (dilation), used with the continuous wavelet transform; τ: A time shift), used with the continuous wavelet transform; W(τ,a): A time-frequency distribution, calculated with the wavelet transform (complex-valued); L2 (R): The set of *square integrable* numbers (being of finite energy); cg: A constant that depends on *g(t)* and *a*; G(ω): The fourier image of the analyzing wavelet *g(t)*; ω: The circular frequency; WBH: A blackman-harris window; T: Duration of the blackman-harris window; ADHD: Attention-deficit-hyperactivity disorder; FPGA: Field programmable gate array; STFT: The short-time fourier transform; WVD: Wigner-ville distribution; Ws(t,f): A time-frequency distribution, calculated with the wigner-ville distribution; Cs(t,f): A time-frequency distribution, calculated with the Choi–Williams distribution; Φ(η,τ): Fernel function of the Choi–Williams distribution.

## Competing interests

The author declares that he has no competing interests.

## Authors’ contribution

The device with which the signals were measured was built several years ago by a team of researchers, of which the author was a part, being responsible for the optoelectronics (lasers and sensors), analog and digital electronics, software for data acquisition, analysis, user interface, archival etc. This device has been extensively evaluated, and the results have been published in different publications, as seen from the references. The time-frequency analysis, subject of this paper, was developed and tested by BG alone. All authors read and approved the final manuscript.
